# Is it feasible to assess self-reported quality of life in individuals who are deaf and have intellectual disabilities?

**DOI:** 10.1007/s00127-020-01957-y

**Published:** 2020-09-14

**Authors:** Johannes Fellinger, Magdalena Dall, Joachim Gerich, Maria Fellinger, Katharina Schossleitner, William Joseph Barbaresi, Daniel Holzinger

**Affiliations:** 1 Research Institute for Developmental Medicine, Johannes Kepler University Linz, Linz, Austria; 2 Konventhospital Barmherzige Brüder, Institut für Sinnes- und Sprachneurologie, Linz, Austria; 3 Division of Social Psychiatry, Medical University of Vienna, Vienna, Austria; 4 Department of Sociology, Johannes Kepler University Linz, Linz, Austria; 5 Division of Developmental Medicine, Department of Pediatrics, Boston Children’s Hospital, Harvard Medical School, Boston, MA USA; 6 Institute of Linguistics, Karl-Franzens University Graz, Graz, Austria

**Keywords:** Quality of life, Intellectual disability, Deafness, Self-report, Sign language

## Abstract

**Purpose:**

There is consensus that Quality of Life (QOL) should be obtained through self-reports from people with intellectual Disability (ID). Thus far, there have been no attempts to collect self-reported QOL from people who are deaf and have ID.

**Methods:**

Based on an established short measure for QOL (EUROHIS-QOL), an adapted easy-to-understand sign language interview was developed and applied in a population (*n* = 61) with severe-to-profound hearing loss and mild-to-profound ID. Self-reports were conducted at two time points (*t*_1_ and *t*_2_), 6 months apart. The Stark QOL, an established picture-based questionnaire, was also obtained at *t*_2_ and three Proxy ratings of QOL (from caregivers) were conducted for each participant at *t*_1_.

**Results:**

Self-reported QOL was successfully administered at both time points for 44 individuals with mild and moderate ID (IQ reference age between 3.3 and 11.8 years).

The self-reports showed sufficient test–retest reliability and significant correlations with the Stark QOL. As anticipated, self-reported QOL was higher than proxy-reported QOL. Test–retest reliability and internal consistency were good for self-reported QOL.

**Conclusion:**

Reliable and valid self-reports of QOL can be obtained from deaf adults with mild-moderate ID using standard inventories adapted to the linguistic and cognitive level of these individuals.

## Introduction

Since the publication of the Convention on the Rights of Persons with Disabilities [1], quality of life has proved to be a useful construct to drive progress towards equity, empowerment, and self-determination [2].

However, obtaining information on Quality of Life directly from people with intellectual disabilities (ID) has been shown to be challenging. Prior studies highlight barriers including language and cognitive function, response bias, and theory of mind [3–6] that are even more relevant in persons who are deaf and have ID [7]. There is consensus that individuals with ID should be directly involved in the measurement of their QOL [8–10]. For this purpose, strategies such as simplifying questions and response options and the use of supportive visuals are required [11]. Proxy ratings are regarded as an important source of additional information, especially in subjects where self-reports are not feasible [8, 9, 12]. Although it is assumed that close relatives or caregivers can provide information that is comparable to the responses which the individual would give [13–18], there are diverging findings on the congruence between self- and proxy-ratings. The majority of studies report higher self-rated QOL scores than proxy-rated QOL scores [10, 19–21], whereas Schwartz and Rabinovitz [17] found the opposite.

Although hearing loss is a common condition in people with ID, with prevalence rates ranging from 30 to 46% [22, 23], we were not able to identify research on assessment methodology of QOL in adults who are deaf and have an intellectual disability. In this population, language deprivation often presents another obstacle to accurate measurement of QOL [24].

In the present study, we aim to develop a reliable and valid procedure to measure self-reported QOL in individuals who are deaf and have ID.

## Methods

### Participants

All participants are enrolled in one of three specialized therapeutic living communities in Austria. These communities are characterized by the constant use of individually adapted sign language and focus on the development of self-determination and social relationships. The entire staff is competent in signed communication; 20% of personnel are themselves deaf. There is a staff-to-client ratio of 1:4. At the time of the study, 61 individuals with severe-to-profound prelingual hearing loss and mild-to-profound ID, aged between 19 and 74 years, have been included in the programs for periods ranging from 6 months to 20 years. Among these 61 people, 13 participate only in the workshop facilities but are not living in the therapeutic residential facilities.

### Measures

#### Intellectual functioning

As cognitive levels of the population varied between mild and profound intellectual disability, two versions of the Snijders-Oomen Non-verbal Intelligence Scale were used: SON-R 6-40 [25] for individuals with an IQ reference age of 6 years or older and the SON-R 2½-7 [26] for the remaining participants. As the SON-R 2 ½-7 does not report IQ scores, IQ reference age is reported for all participants.

#### QOL measures

All Quality-of-Life measures used are based on the EUROHIS-QOL 8-item index (European Health Interview Surveys [27]). It consists of eight questions that are also included in both the WHOQOL-100 questionnaire [28–30] and the WHOQOL-BREF (an abbreviated version of the WHO-QOL 100 with 26 items; [30–32]. For the WHOQOL-BREF, a sign language version has been previously developed that is designed for full self-administration and tested in a large deaf population [33].

The EUROHIS-QOL includes two questions representing each of the four domains (physical, psychological, social, and environmental) and produces an overall QOL score. Responses are given on a five-point Likert scale. The EUROHIS-QOL score is computed as the mean score across the eight items on the measure, with scores ranging from 1 (worst QOL) to 5 (best QOL) for each item. The EUROHIS-QOL has been assessed across various settings and countries [27]. A general population study in Germany showed good reliability as well as construct validity [34].

#### Self-reports—EUROHIS-QOL

For our study sample, the EUROHIS-QOL was translated and adapted into an easy-to-understand sign language version (EUROHIS-QOL ESL). The EUROHIS-QOL was adapted to the needs of individuals with ID and limited sign language skills. We followed the international suggestions for translation of quality-of-life measures [35, 36] and those for easy-to-understand (easy-to-read) language [37, 38], as well as recommendations for translation from spoken into signed languages [39]. A deaf professional working in education and care and a sign competent neuropsychiatrist reviewed the contents of the German EUROHIS-QOL and of the sign language version of the WHOQOL-BREF [33], and developed a first draft of an easy-to-understand sign language version. A revised second version included input from a linguist, a psychologist, and four care professionals, all fluent in Austrian sign language. This version was video recorded with a native signer, back translated by a professional Austrian sign language interpreter, and piloted with three participants by presenting the videos of the signed questions. Questions were answered using a five-point visually based Likert scale with smileys. As recommended by others [4, 40], the Likert scale was explained and training questions were offered immediately before the questions, to ensure that respondents understood how to respond to the items. This pilot showed that, contrary to prior experience in a general population of individuals who are deaf [33], computer-based self-administration was not possible in this population of persons with deafness and ID. Therefore, the EUROHIS-QOL ESL was subsequently administered as a standardized face-to-face interview. Interviewers were provided a video template and a written interview guideline to ensure the highest possible standardized administration of the EUROHIS-QOL ESL and to prevent translations on the fly [41]. This interview guideline offered the flexibility to add scripted examples to maximize understanding of the contents and to ensure higher response rates. This approach has also been used in the previous research studies [4, 42]. As with the EUROHIS-QOL, the EUROHIS-QOL ESL score is computed as the mean score across the eight items, ranging from 1 (worst QOL) to 5 (best QOL). The interviewers rated the participants’ comprehension of each question on a three-point Likert scale [good (three points), uncertain (two points), and no comprehension (one point)]. The maximum possible score for comprehension of the instrument was, therefore, 24 points. Participants with comprehension scores less than 16 points were excluded from this study. We identified a subgroup of participants with good comprehension (22–24 points, maximum of two questions rated as uncertain) to evaluate the instrument in persons deemed capable of understanding the EUROHIS-QOL ESL items.

#### Proxy-ratings—EUROHIS-QOL

Professional caregivers were asked to answer questions on the proxy questionnaire (EUROHIS-QOL) from the participants’ perspective, as described by Pickard and Knight [43] and McPhail et al. [44]. For participants living at the therapeutic community, there are three separate proxy ratings (proxy 1 and proxy 2 from the therapeutic residential facility and proxy 3 from the workshop facility). For the 13 participants, who were only involved in the workshop facilities, only the rating from the caregiver (proxy 3) in the workshop facility was obtained. In total, 66 staff members (73% female; mean age 41 years) completed the EUROHIS-QOL proxy questionnaire.

#### Validation of QOL measures

For the validation of the EUROHIS-QOL ESL the Stark Quality of Life Questionnaire (Stark QOL; [45] was administered. The Stark QOL uses short questions that were translated into easy-to-understand sign language, and descriptive pictures as response options. Three items of the Stark QOL with respect to mood, energy, and social contact were selected; these three items can be combined into the Stark QOL mental component with a score range from 0 (worst QOL) to 100 (best QOL). One possible limitation of this approach is the risk of mono- or common-method bias [46]. This may inflate the correlation between EUROHIS-QOL ESL and the Stark measure by applying a similar method of data collection for both constructs. Higher risks for method bias are assumed for measures addressing cognitive and emotional states [47] where acquiescence or the use or avoidance of extreme response categories [48] has been observed.

Therefore, an additional experimental validation approach was employed, using a different mode of data collection that we term “Light response”. Respondents rated their general well-being by adjusting the brightness of a light bulb with the help of a five-level controller to express their perception of their QOL, where complete darkness (level 1) corresponded to the lowest QOL and the brightest level (level 5) corresponded to the best QOL.

### Procedure

Data collection took place between September 2017 and March 2018. To obtain test–retest reliability, the self-report interviews were conducted twice (*t*_1_ and *t*_2_), with 6 months in between, by one care professional per site (two deaf and one hearing fluent in sign language). The interviewers were not directly involved in care and did not act as a proxy in this study, but knew the participants well enough to successfully conduct the interviews. The non-involvement in care was considered important, in order for the participants not to feel pressured to give answers that they thought would be satisfactory for the interviewer. Nevertheless, it was also important for the interviewers to understand the participants’ way of communication. Validation measures were obtained at *t*_2_ only. Proxy ratings of QOL were conducted at *t*_1_ only.

### Statistical analysis

Intraclass correlation coefficient (ICC) was used to estimate test–retest reliability of self-reported EUROHIS-QOL ESL and agreement between self- and proxy ratings. As the proxy raters in our study were fixed (e.g., not based on a random sample) and the agreement is assumed for any randomly selected participant, ICC’s were computed on the basis of a two-way mixed ANOVA model (ICC model three, [49]. Moreover, as our analysis focuses on the comparison of one rating per rater for each case, the ICC form is one (ICC for single measures). Therefore, the computed coefficients are of the type ICC (3, 1) [50].

ICC analysis allows differentiation of the degree of agreement into “absolute agreement” (identical rating) or “consistency agreement” (i.e., higher ratings of one rater correspond to higher ratings of the other and vice versa, but both ratings are not necessarily identical). A high ICC based on consistency agreement—which is in most cases identical to Pearson's correlation—indicates high consistency of ratings which is invariant with respect to linear transformations. Typically, the ICC based on absolute agreement will be smaller than the ICC based on consistency agreement. Consequently, larger differences between both types of ICCs indicate that systematic under- or overestimation of one rater compared to the other. ICC (3, 1) is also used as an estimator of test–retest reliability [50, p.131]. Moreover, reliability of proxy- and self-reports (separately computed for measures administered at *t*_1_ and *t*_2_) is estimated on the basis of internal consistency (Cronbachs Alpha). According to Fleiss [51] and Trevethan [50], ICC values < 0.40 can be classified as poor and values between 0.40 and 0.75 as fair-to-good agreement for non-clinical applications. Values of Cronbachs Alpha > 0.7 are classified as sufficient and values > 0.8 as good internal consistency [52].

*T *test for paired samples was used to compare mean QOL scores of self- and proxy-ratings.

### Ethics

The study was approved by the ethics committee at the hospital Barmherzige Brüder Linz, Austria.

## Results

### Participants

Among the 61 eligible persons, 12 persons could not participate at either time point due to a lack of basic understanding. For another eight persons, responses were obtained at only one time point due to organizational or personal reasons (e.g., longer hospital stays and refusal to participate). Complete data at both time points are available for 41 individuals (67% of total sample), 25 of whom (41% of total sample) were in the subgroup of those with good comprehension. In total, there were 47 (77%) complete responses to the EUROHIS-QOL ESL at *t*_1_ and 43 (70%) complete responses at *t*_2_.

IQ reference age is available for 57 persons. The missing data are from persons who either refused IQ testing or for whom testing resulted in an IQ reference age below 2 years. Descriptive information regarding age, sex, and IQ reference age of the study population is given in Table 1. Comparison of the mean EUROHIS-QOL ESL scores of those individuals (1) without complete self-reports, (2) complete self-reports but limited comprehension, and (3) complete self-reports and good comprehension shows significant differences with respect to IQ reference age.

**Table 1:** Demographics

	Total (*n* 61)	*n*	Subsample with complete self-reports at both time points (*n* 41)	*n*	Subsample with good comprehension (*n* 25)	*n*	*p* value Anova^a^
Age in years *M* (SD)	45.67 (18.31)	61	46.93 (18.07)	41	45.88 (17.22)	25	0.657
Sex (female %)	37.7	61	43.9	41	44.0	25	0.372
IQ reference age in years *M* (SD)	6.45 (2.14)	57	6.87 (2.08)	41	7.41 (1.80)	25	0.006

^a^Anova comparing age, sex and IQ reference age (means) of excluded individuals, individuals with complete responses but limited comprehension, and individuals with complete responses and good comprehension

As is apparent from Table 1, the IQ reference age level of the subpopulation with complete self-reported data (*n* = 41) is higher compared to the non-participants (*n* = 16). The range of the IQ reference age in the total population is between 2.8 and 11.8 years, whereas the range in the subpopulation with complete data is between 3.3 and 11.8. Being a member of the subgroup with complete self-reported data is significantly correlated with IQ reference age (*r* = 0.32; *p* = 0.017). Good comprehension of the responders is also significantly correlated with IQ reference age (*r* = 0.33; *p* = 0.036). Differences in age and sex between the subsample of responders with complete data, as well as the subsample with good comprehension compared to the other participants are not significant (*p* > 0.05). For 9 of the 12 participants who could not participate due to a lack of basic understanding, mean IQ reference age was 4.28 years (SD = 0.85), mean chronological age was 45.7 years (SD = 19.3; *n* = 12), and 25% were female.

Results for the QOL measures and the validation measures (Stark QOL and Light Response) for the sample with self-reports available for both time points are shown in Table 2.

**Table 2:** Descriptive information for the QOL and validation measures

	Subsample with complete self-reports at both time points	*n*	Subsample with good comprehension	*n*	*p* value^a^
EUROHIS-QOL ESL Self-report score *t*_1_ *M* (SD)	4.23 (0.65)	41	4.37 (0.65)	25	0.086
EUROHIS-QOL ESL Self-report score t_2_ M(SD)	4.27 (0.66)	41	4.30 (0.73)	25	0.699
EUROHIS-QOL score Proxy 1 *t*_1_ *M* (SD)	3.88 (0.61)	29	3.83 (0.67)	18	0.648
EUROHIS-QOL score Proxy 2 *t*_1_ *M* (SD)	3.70 (0.75)	29	3.58 (0.79)	18	0.283
EUROHIS-QOL score Proxy 3 *t*_1_ *M* (SD)	3.82 (0.45)	41	3.85 (0.43)	25	0.571
EUROHIS-QOL score Proxy mean *t*_1_ *M* (SD)	3.81 (0.52)	41	3.83 (0.54)	25	0.835
Stark QOL score self-report *t*_2_ *M* (SD)	73.64 (29.75)	41	71.10 (29.77)	25	0.502
Light Response	4.11 (1.21)	36	3.86 (1.36)	22	0.127

^a^*p* value for differences of means between the group with good versus limited questionnaire comprehension (*t *test)

QOL scores and scores on validation measures did not differ between participants with good comprehension versus those with limited comprehension (Table 2).

### Reliability estimates of EUROHIS-QOL ESL

Table 3 shows reliability estimations for self-reported QOL data (test–retest and internal consistency). Test–retest reliability based on consistency measures (ICC for consistence) as well as based on absolute agreement of QOL scores between both time points is good (> 0.7). Similarly, with respect to Cronbachs Alpha, sufficient internal consistency at both time points with values higher than 0.7 is estimated. Reliability estimates for the subsample with good comprehension (≥ 0.8) indicate good reliability.

**Table 3:** Reliability estimation of self and proxy reports

	Total sample	Subsample good comprehension
Self-reports: test–retest		
ICC (3,1), consistency	0.75	0.83
ICC (3,1), absolute	0.75	0.83
Self reports: internal consistency		
Cronbachs Alpha *t*_1_	0.78	0.81
Cronbachs Alpha *t*_2_	0.78	0.80
Proxy reports: agreement		
Mean ICC(3,1), consistency	0.61	
Mean ICC(3,1), absolute	0.61	
Proxy reports: internal consistency		
Mean Cronbachs alpha	0.80	

### Reliability of proxy measures

Table 3 also shows ICC measures for consistency and absolute agreement between the proxy-rated QOL scores. The mean ICC coefficient for consistency as well as for absolute agreement of all three pairs of proxy ratings was 0.61 (range between 0.56 and 0.68) which can be classified as fair-to-good agreement. Also, sufficient to good internal consistency of the three proxy measures was confirmed (mean Cronbachs Alpha across all three proxy ratings is 0.80 with a range between 0.72 and 0.87).

### Estimation of validity

Table 4 shows the correlations of self- and proxy measures with the Stark-questionnaire score and the Light response (both measured at *t*_2_). Significant positive correlations between self-reported EUROHIS-QOL ESL at both time points and Stark-questionnaire score are confirmed. Correlations are somewhat higher for self-reports measured at *t*_2_ and for the subsample with good comprehension. In contrast, none of the proxy QOL measures, taken at time point 1, are significantly correlated with the Stark QOL measure. The correlations between self-reported EUROHIS-QOL ESL scores at *t*_1_ and the Light Response are not significant. However, at *t*_2_, a marginally significant positive correlation (*p* < 0.10) for the total sample and a significant positive correlation (*p* < 0.05) for the subsample with good comprehension were found. As with the Stark QOL, no significant correlations between Light Response and the proxy QOL scores were found.

**Table 4:** Correlations of self- and proxy reports with Stark QOL and light response

	Stark QOL				Light response			
	Total sample with complete responses at both time points		Subsample with good rated comprehension		Total sample with complete responses at both time points		Subsample with good rated comprehension	
	*r* (*p*)	*n*	*r* (*p*)	*n*	*r* (*p*)	*n*	*r* (*p*)	*n*
Qol8 self-report *t*_1_	0.33 (0.038)	41	0.52 (0.007)	25	0.16 (0.343)	36	0.20 (0.376)	22
Qol8 self-report *t*_2_	0.42 (0.007)	41	0.71 (< 0.001)	25	0.31 (0.067)	36	0.44 (0.042)	22
Qol8 proxy 1	0.23 (0.233)	29	0.31 (0.216)	18	0.11 (0.596)	25	0.26 (0.348)	15
Qol8 proxy 2	0.27 (0.151)	29	0.51 (0.031)	18	− 0.01 (0.963)	25	0.04 (0.882)	15
Qol8 proxy 3	0.17 (0.289)	41	0.43 (0.034)	25	− 0.06 (0.740)	36	0.07 (0.772)	22

### Agreement between self-reports and proxy measures

Table 5 shows the results regarding self-proxy agreement using ICC based on consistency and absolute agreement. At *t*_1_, agreement is only significant for proxy 2 (*p* < 0.05) and marginally significant for proxy 1 and proxy 3 (*p* < 0.10). All ICC values are smaller than 0.4, suggesting poor agreement between proxy scores and self-reports collected at *t*_1_. In contrast, agreements between the subsample with good comprehension at *t*_1_ and all proxy measures are significant (*p* < 0.05) with ICC consistency values ≥ 0.4. Therefore, fair-to-good consistency agreement is suggested for this subpopulation. However, as self-rated QOL scores are systematically higher compared to proxy-rated QOL, absolute agreement is lower and ICCs based on absolute agreement still fall beyond the threshold of 0.4 for fair-to-good agreement in this subsample.

**Table 5:** Agreement between self- and proxy-reports

	Proxy 1, self	Proxy 2, self	Proxy 3, self	Mean ICC
*t*_1_ Total sample of responders				
ICC (3, 1) consistency	0.31	0.39	0.23	0.31
ICC (3, 1) absolute	0.26	0.29	0.19	0.25
* p* (ICC)	0.051	0.017	0.068	
Mean difference (*p*)	0.41 (0.007)	0.60 (< 0.001)	0.41 (0.001)	
* n*	29	29	41	
*t*_1_ Subsample good comprehension				
ICC (3, 1) consistency	0.42	0.42	0.40	0.41
ICC (3, 1) absolute	0.34	0.28	0.28	0.30
*p* (ICC)	0.036	0.038	0.021	
Mean difference (*p*)	0.52 (0.008)	0.77 (0.001)	0.52 (< 0.001)	
*n*	18	18	25	
*t*_2_ Total sample of responders				
ICC (3, 1) consistency	0.45	0.59	0.39	0.48
ICC (3, 1) absolute	0.37	0.42	0.30	0.36
*p* (ICC)	0.006	< 0.001	0.005	
Mean difference (*p*)	0.45 (0.001)	0.64 (< 0.001)	0.45 (< 0.001)	
*n*	29	29	41	
*t*_2_ Subsample good comprehension				
ICC (3, 1) consistency	0.49	0.55	0.46	0.50
ICC (3, 1) absolute	0.43	0.40	0.36	0.40
*p* (ICC)	0.016	0.007	0.009	
Mean difference (*p*)	0.43 (0.021)	0.68 (0.001)	0.45 (0.001)	
*n*	18	18	25	

*p* (ICC): *p* value for ICC’s; mean difference: mean of QOLself and QOLproxy (*p* value based on *t* test for paired samples)

In contrast, all self-proxy agreements based on self-reports measured at *t*_2_ are significant (*p* < 0.05). The mean value of ICCs across the proxies based on consistency agreement is > 0.4 with only small differences between the total sample and the subpopulation with good comprehension. Again, smaller values for the ICCs based on absolute agreement compared to consistency agreement are observed, although even the mean value of absolute agreement reaches the threshold of 0.4 for the subpopulation with good comprehension. The mean absolute agreement for the total sample falls beyond the threshold of 0.4.

In sum, poor agreement between self and proxy measures was found for self-reports collected at *t*_1_ with respect to the total sample, whereas self-reports of the subsample with good comprehension at t_1_ and self-reports of the total sample at *t*_2_ show fair-to-good consistency agreement.

As the ICC’s of absolute self-proxy agreement were shown to be lower compared to ICCs based on consistency agreement, systematic mean differences of self-reported QOL compared to proxy-rated QOL could be expected, which is also apparent from the descriptive information given in Table 2.

Table 5 shows the *p* values for the differences of mean QOL scores between self-rating and proxy rating. The self-rated QOL scores at both time points are significantly higher compared to all three proxy ratings (*p* < 0.05). Further analyses (not shown in Table 5) reveal that no significant differences in the mean QOL scores within the three proxy ratings are observed (*p* = 0.327–0.667). Similarly, no significant differences in the mean scores of self-reported QOL between both time points are found (*p* = 0.573).

To summarize, mean self-rated QOL is consistently higher compared to proxy ratings of QOL. QOL scores of self-reports are stable across both time points, and no significant score differences are observed between the three proxy raters.

## Discussion

Many studies reporting on the Quality of Life of people with intellectual disabilities draw upon proxy ratings versus asking the individuals about their self-reported QOL. Therefore, the goal of this study was to develop an easy-to-understand sign language version of a standardized quality of life inventory (EUROHIS-QOL), adapted to the visual communication needs of people with prelingual deafness and ID (EUROHIS-QOL ESL).

The easy-to-understand sign language version EUROHIS-QOL ESL can be administered in a time-efficient manner and with high acceptance by individuals who are deaf and have mild-to-moderate ID. 61% of the people who participated at both time points were rated as having good comprehension by the interviewers. Our finding of increased responsiveness and comprehension with higher IQ is consistent with the previous research [53].

Based on internal consistency as well as on the test–retest method, sufficient reliability (> 0.7) of the EUROHIS-QOL ESL measure was confirmed for the total sample of responders. Reliability in the subsample with good questionnaire comprehension was high (> 0.8). With respect to validity, significant correlations between self-rated QOL and the Stark-questionnaire measure were confirmed for the total sample of responders at both time points. In contrast, none of the three proxy QOL ratings were significantly correlated with respondents’ scores on the Stark measure in the total sample. As with reliability, higher validity was estimated for the subsample with good comprehension. Moreover, two out of the three proxy ratings were significantly correlated with respondents’ scores on the Stark measure in the subsample with good comprehension. To avoid a possible inflation of correlations due to common- or mono-method bias, an alternative experimental approach, the “Light Response” was evaluated. Overall, only weak and mostly insignificant associations between QOL measures and the Light response were found. This might be attributed to the higher cognitive demands in understanding the metaphor between brightness of light and well-being. Nevertheless, a marginally significant correlation between the Light response and the EUROHIS-QOL ESL scores measured at *t*_2_ and a significant correlation for the subsample with good questionnaire comprehension at *t*_2_ was confirmed, whereas proxy QOL measures were not significantly correlated with the Light response.

With respect to the consistency agreement between self-reported and proxy-reported QOL, poor concordance (ICC < 0.4) was found for the self-ratings of the total sample at *t*_1_. However, fair-to-good agreement (ICC ≥ 0.4) was confirmed for the subsample with good comprehension. Regarding the total sample of responders, fair-to-good mean consistency agreement for self-ratings at *t*_2_ and proxy-reported QOL measured was confirmed. Again, for measures at *t*_2,_ consistency was slightly higher in the subsample with good questionnaire comprehension, but the difference between those with good comprehension and the total sample was less pronounced compared to the measures taken at *t*_1_.

Unlike consistency-based agreement between self and proxy ratings, absolute agreement was considerably weaker and, except for the subsample with good comprehension at *t*_2_, classified as poor on the basis of thresholds suggested by Trevethan [50]. Hence, although there is a systematic significant relationship between self- and proxy ratings, the results suggest that this concordance does not necessarily mean absolute agreement of self- and proxy ratings. Our results confirm that self-reported QOL was significantly higher compared to proxy-rated QOL, which is consistent with the results of most of previous studies [10, 19–21]. Nevertheless, it cannot be ruled out, for example, that the systematic difference between self- and proxy-rated QOL is due to acquiescence bias or a higher tendency of individuals with ID to choose extreme or socially desirable answer categories [54]. However, it is also likely that proxies rate QOL of individuals with disabilities based on their own values and expectations, resulting in an underestimation of the proxy-rated QOL [12, 19, 55]. Moreover, proxies may predominately base their estimation on manifest (e.g., bodily conditions or social activities) or communicated information provided by the individuals with disability, which may be poor indicators of the subjective QOL of the rated individuals.

Our results showed somewhat higher estimated validity and consistency agreement for measures taken at *t*_2_ compared to *t*_1_. An improvement of data quality associated with repeated measurement is not uncommon and known as a specific type of a panel conditioning effect through cognitive stimulation [56]. Hence, a possible interpretation could be that individuals with ID benefit from repeated survey administration, by learning how to deal with the unfamiliar survey procedure and enhancement of the cognitive process of answering specific types of questions. This interpretation would be consistent with van de Vijver and Poortinga [48], who noted that especially “individuals with little test experience can be expected to gain more from repeated test administration”. From this perspective, it could be expected that data quality may be improved either through a combination of measures taken from repeated administration, or by implementing practice trails before the final administration.

## Limitations

First, the presented results are based on a small sample size. Although large-scale research in populations with deafness and ID is difficult, replications with larger sample sizes will be needed to confirm the findings from this study. Larger sample size would also be needed to better understand variations in QOL responses and measurement in the highly heterogeneous population of persons who are deaf and have ID. To counteract this limitation, the sample was divided into three groups based on the ability to respond and the level of the comprehension of the questions. However, these subsamples were not large enough to enable more thorough analyses. Not all measures were collected at both time points. Due to the logistical challenges noted previously, proxy ratings were only administered at time point 1. However, the previous research suggests that Quality of Life is a rather stable parameter [57], and therefore, comparisons of self-rated QOL at *t*_2_ and proxy-rated QOL at *t*_1_ with a time gap of 6 months seem to be a justified. Questionnaire comprehension was rated by a single interviewer. For future studies, it would be beneficial to have two independent raters, which would enable the estimation of interrater-reliability.

## Conclusion

This study was based on the principle that people with ID should have the opportunity to self-report their quality of life, which also applies to people who are deaf. The QOL ESL, with its simple linguistic structure and choice of basic vocabulary, could be administered to people with deafness and mild-to-moderate ID, and provided reliable and valid results. This opens opportunities to include also individuals who are deaf and have ID themselves into dimensions of quality-of-life research.

## Data Availability

All data and materials are stored anonymously with the authors.
